# The Influence of Chemical Composition of Commercial Lemon Essential Oils on the Growth of *Candida* Strains

**DOI:** 10.1007/s11046-013-9723-3

**Published:** 2014-01-12

**Authors:** M. Białoń, T. Krzyśko-Łupicka, M. Koszałkowska, P. P. Wieczorek

**Affiliations:** 1 Faculty of Chemistry, University of Opole, Oleska 48, 45-052 Opole, Poland; 2 Independent Department of Biotechnology and Molecular Biology, Faculty of Natural and Technical Science, University of Opole, Kominka 6A, 45-035 Opole, Poland

**Keywords:** *Candida albicans*, Antifungal, Lemon essential oils, GCMS

## Abstract

*Candida* yeasts are saprophytes naturally present in the environment and forming colonies on human mucous membranes and skin. They are opportunistic fungi that cause severe and even fatal infections in immunocompromised individuals. Several essential oils, including eucalyptus, pine, cinnamon and lemon, have been shown to be effective against *Candida* strains. This study addresses the chemical composition of some commercial lemon essential oils and their antifungal potential against selected *Candida* yeast strains. Antifungal potential and minimum inhibitory concentrations were determined for six commercial lemon essential oils against five *Candida* yeast strains (*Candida albicans* 31, *Candida tropicalis* 32, *Candida glabrata* 33, *Candida glabrata* 35 and *Candida glabrata* 38). On the basis of the GCMS analysis, it was found that the tested lemon essential oils had different chemical compositions, but mostly, they contained almost exclusively terpenes and oxygenated terpenes. The tests show that antifungal potential of lemon essential oils against *Candida* yeast strains was related to the high content of monoterpenoids and the type of *Candida* strains. From six tested commercial oils, only four (ETJA, Vera-Nord, Avicenna-Oil and Aromatic Art) shows antifungal potential against three *Candida* species (*C. albicans*, *C.* *tropicalis* and *C.* *glabrata*). Vera-Nord and Avicenna-Oil show the best activity and effectively inhibit the growth of the *C. albicans* strain across the full range of the concentrations used. Our study characterises lemon essential oils, which could be used as very effective natural remedies against candidiasis caused by *C. albicans*.

## Introduction

The interest in natural antimicrobial preparations, such as extracts and essential oils [1–3], has significantly grown in recent years. The biological activity and efficacy of essential oils depend on the quality and quantity of the active substance contained in the raw material [4, 5], which in turn depends on the plant variety, genus and quality, growing conditions (climate, soil and fertilisation), time of harvest as well as the way the essential oil is produced and stored. The inhibition of the growth of bacterial and fungal strains is related to volatile compounds contained in essential oils [6] and the sensitivity of microorganisms.

*Candida* yeasts are saprophytes naturally present in the environment and forming colonies on human mucous membranes and skin. They are opportunistic fungi that cause severe and even fatal infections in immunocompromised individuals. Several essential oils, including eucalyptus, pine, cinnamon and lemon, have been shown to be effective against *Candida* strains [3, 5, 7]. However, the reports on the biological activity of lemon essential oil are ambiguous. Some literature data recommend it as highly effective, while other reports that the effects of its use are quite ordinary [7, 8]. Differences in potential fungicidal properties of lemon essential oils may be due to variable qualitative and quantitative composition of individual essential oils and are related to the development stage of the fruit prior to extraction, fruit condition and quality as well as plant growing conditions. According to Fisher and Phillips [9], the content of volatile substances in lemon essential oils may range from 85 to 99 %. The qualitative composition—proportions between the content of particular monoterpenes, sesquiterpenes and their oxygenated derivatives—changes as well [10, 11]. The highest concentration of volatile substances in lemon essential oils is observed in oils produced from medium-ripe fruit [12]. Citrus essential oils are among the oils generally regarded as safe (GRAS) by the US Food and Drug Administration [13].

The aim of the study was to analyse the chemical composition of commercial lemon essential oils (*Citrus limonum* L.) available on the European market and to evaluate their antifungal potential against selected *Candida* yeast strains.

## Materials and Methods

### Research Material

- *Candida* yeast strains consist of the following: *Candida albicans* 31 (C31), *Candida tropicalis* 32 (C32), *Candida glabrata* 33 (C33), *Candida glabrata* 35 (C35) and *Candida glabrata* 38 (C38). *Candida* yeast strains were isolated from bronchial secretions and came from the Collection of Polish National Medicines Institute in Warsaw. The yeasts were incubated on medium YPG slants (2 % Yeast Extract; 2 % Peptone; 2 % Glucose) [14] at 30 °C for 48 h, washed with Tween 80 water and standardised. The 2 × 10^5^ CFU ml^−1^ yeast suspension was used for tests.
- Lemon essential oils (*Citrus limonum L*) from various countries available on the Polish market: ETJA (ETJA, Elbląg. Poland), Vera-Nord (“VERA-NORD” S.C., Legionowo, Poland), Avicenna-Oil (Avicenna-Oil^®^, Wrocław, Poland), Dufti by Gies (GIES Kerzen GmbH, Glinde, Germany), Aromatic Art (Müller Kerzenfabrik, Straelen, Germany) and Croce Azzurra (CROCE AZZURRA S.N.C., Brescello, Italy) were tested. The activity of essential oil solutions (in water with 0.05 % Tween 80) at the final concentration of 0.6, 1.0, 1.6, 2.0, 2.6 and 3.0 % was tested.

### Gas Chromatography/Mass Spectrometry (GC/MS) Analysis

The analysis of essential oils samples was performed on Gas Chromatograph HP 6890 coupled with mass spectrometry HP 5973A (Hewlett–Packard). Non-polar capillary column HP-5MS (5 % diphenyl, 95 % dimethylpolysiloxane), with a length of 30 m, internal diameter 0.25 mm id and film thickness of 0.25 μm, was used. Helium was used as a carrier gas, and its flow was 1.5 ml min^−1^. Analyses were performed in the temperature range 60–280 °C, and the heating rate was 10 °C min^−1^. The injection volume was 1 μl of 1:50 (v/v) solution of citrus essential oils in dichloromethane. The analysis was repeated three times for each sample.

### Essential Oil Screening by the Cylinder-Plate Diffusion Method

The bactericidal activity of lemon essential oils with the varying content of terpene compounds against *Candida* yeast was evaluated by the cylinder-plate diffusion method in four repetitions [15]. The media were inoculated by means of 1-ml standardised yeast suspension (2 × 10^5^ CFU ml^−1^). The final concentration of essential oil (in water with 0.05 % Tween 80) was 0.6 %, 1.0, 1.6, 2.0, 2.6 and 3.0 %. Water with 0.05 % Tween 80 was used as a control. The plates were incubated at 30 °C for 48 h. The results are given as the mean value of the diameter of the inhibition of microbial growth in mm. The following criteria were assumed: inhibitory effect—when no growth around the wells is observed; neutral activity—the growth stopped at the edge of the wells; stimulation—more intense growth around the wells.

### Determination of the Minimum Inhibitory Concentration (MIC)

The minimum concentration of essential oils inhibiting the growth (MIC) of the tested yeast strains was determined by the tube dilution method in a liquid YPG medium containing essential oils at the final concentrations of 0.6, 1.0, 1.6, 2.0, 2.6 and 3.0 %. The culture of the tested strains in a liquid YPG medium (without essential oils) was used as a control. The media were inoculated by means of 1 ml standardised yeast suspension (2 × 10^5^ CFU ml^−1^). After 48 h of incubation at 30 °C, the number of viable cells of microorganisms was determined by the cultivation (on a solid YPG medium) method of ten-fold (10^−1^–10^−6^) dilutions of the culture in water with 0.05 % Tween 80. After incubation, the number of alive microorganisms (not sensitive to the essential oil) was determined. The minimum inhibitory concentration (MIC) was determined for each essential oil concentration and type.

## Results

**Fig. 1:**
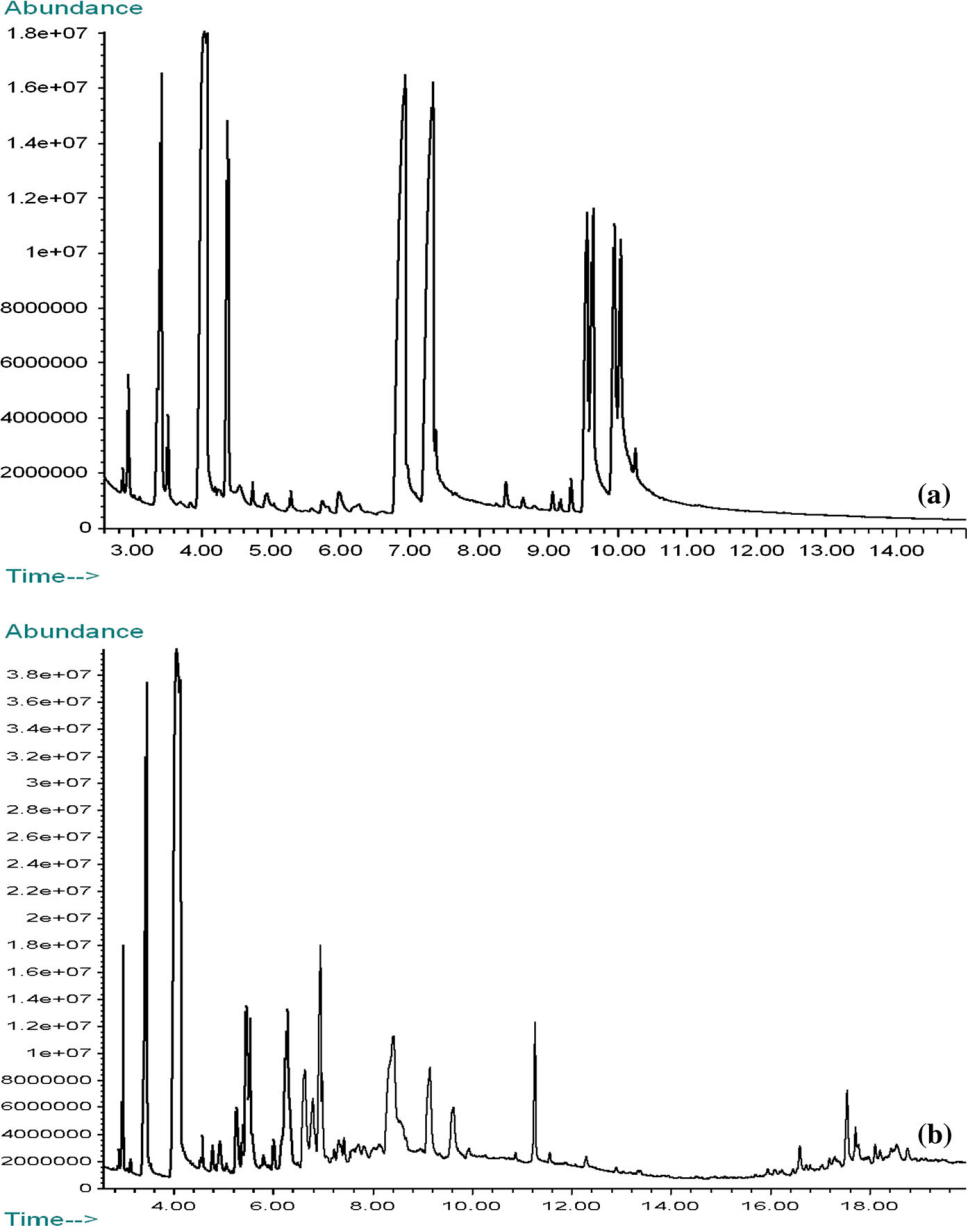
Gas chromatogram of most effective oils: **a** Vera-Nord lemon essential oil, **b** Avicenna-Oil lemon essential oil

**Fig. 2:**
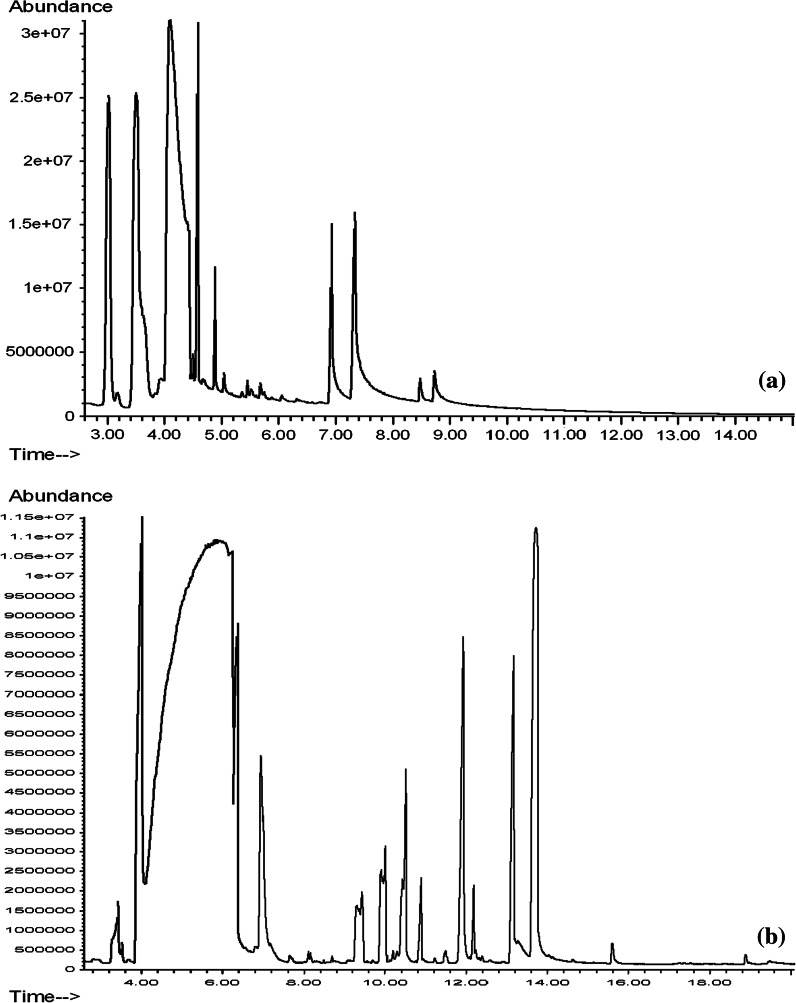
Gas chromatogram of effective oils: **a** ETJA lemon essential oil, **b** Aromatic Art lemon essential oil

**Fig. 3:**
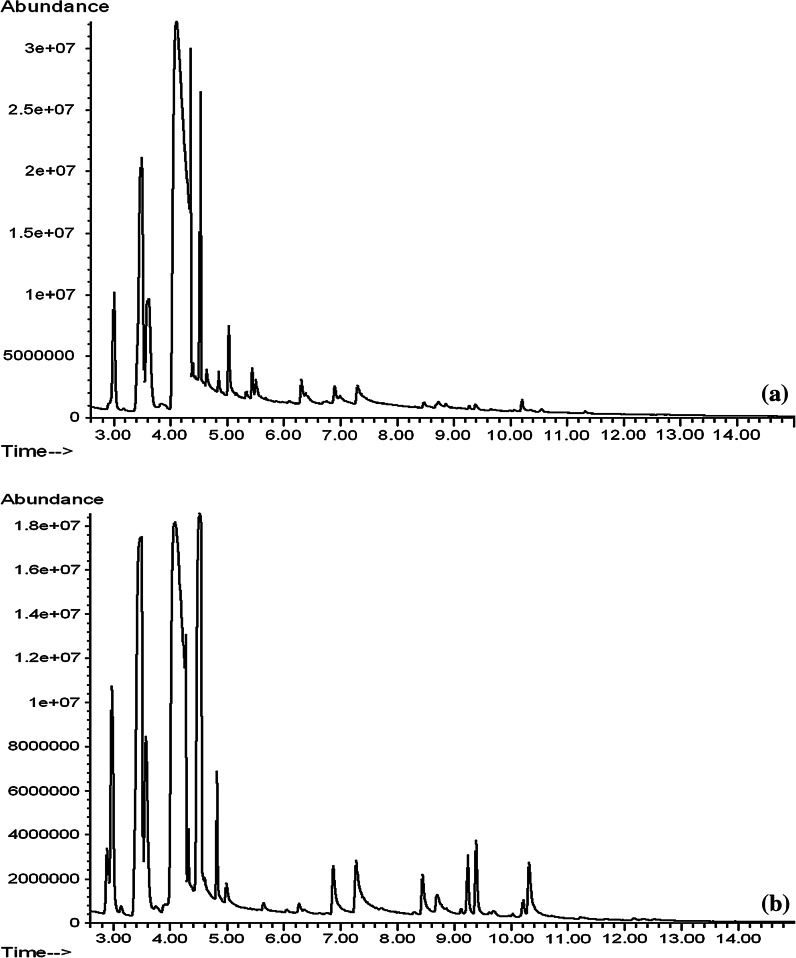
Gas chromatogram of ineffective oils: **a** Dufti by Gies lemon essential oil, **b** Croce Azzurra lemon essential oil

On the basis of the GCMS analysis, it was found that the tested lemon essential oils had different chemical compositions, but, except the Aromatic Art essential oil, they contained almost exclusively terpenes and oxygenated terpenes. The content of monoterpenes in the tested essential oils is shown in Table 1, monoterpenoids in Table 2 and sesquiterpenes, oxygenated sesquiterpenes and other compounds of the six tested essential oils in Table 3. Figures presented gas chromatograms of tested lemon essential oils: Fig. 1a Vera-Nord, Fig. 1b Avicenna-Oil, Fig. 2a ETJA, Fig. 2b Aromatic Art, Fig. 3a Dufti by Gies, Fig. 3b Croce Azzurra.

**Table 1:** Monoterpenes in lemon essential oils

	ETJA		Vera-Nord		Avicenna-Oil		Dufti by Gies		Croce Azzurra		Aromatic Art	
	Area (%)	SD	Area (%)	SD	Area (%)	SD	Area (%)	SD	Area (%)	SD	Area (%)	SD
*Aliphatic monoterpenes*												
ß-myrcene	4.11	±0.063	1.46	±0.046	0	0	6.94	±0.093	5.42	±0.026	0.37	±0.017
trans-ocimene	0.13	±0.025	0.49	±0.049	0	0	0	0	0	0	0	0
*Monocyclic monoterpenes*												
ß-felandrene	0	0	0	0	0	0	0.40	±0.013	15.03	±0.092	0	0
limonene	48.27	±0.048	23.39	±0.054	42.03	±0.124	63.20	±0.007	38.50	±0.074	22.42	±0.017
α-terpinene	0.40	±0.121	0.12	±0.016	0	0	0	0	0.14	±0.032	0	0
γ-terpinene	4.85	±0.057	5.87	±0.015	0	0	4.78	±0.087	0.16	±0.013	0	0
terpinolene	1.19	±0.027	0.30	±0.038	0	0	0.34	±0.046	0.04	±0.004	0	0
*Bi- and tricyclic monoterpenes*												
bornylene	0	0	0	0	0.49	±0.048	0	0	0	0	0	0
camphene	0.35	±0.038	0	0	0.17	±0.014	0	0	0	0	0	0
3-carene	0	0	0	0	0	0	0.16	±0.027	0	0	0	0
α-pinene	11.06	±0.135	1.44	±0.012	3.42	±0.026	3.61	±0.110	5.56	±0.082	0.16	±0.024
ß-pinene	15.14	±0.038	8.93	±0.035	15.15	±0.057	14.31	±0.163	19.98	±0.097	2.21	±0.068
3-thujene	0	0	0.30	±0.036	0.22	±0.013	0.33	±0.037	1.25	±0.067	0	0
sabinene	0	0	0.65	±0.093	0	0	0	0	0	0	0.10	±0.026
tricyclene	0.21	±0.037	0	0	0	0	0	0	0	0	0	0
*Monoterpenes*	*85.70*		*42.96*		*61.49*		*94.07*		*86.07*		*25.27*	

*SD* standard deviation

**Table 2:** Oxygenated monoterpenes in lemon essential oils

	ETJA		Vera-Nord		Avicenna-Oil		Dufti by Gies		Croce Azzurra		Aromatic Art	
	Area (%)	SD	Area (%)	SD	Area (%)	SD	Area (%)	SD	Area (%)	SD	Area (%)	SD
*Oxygenated aliphatic monoterpenes*												
citral (mix of isomers)	0	0	0.23	±0.009	0.70	±0.017	1.06	±0.028	2.63	±0.034	0	0
trans-citral	7.14	±0.067	15.52	±0.042	0	0	0	0	0.16	±0.015	0.36	±0.031
cis-citral	4.30	±0.140	19.41	±0.021	0	0	0.30	±0.060	1.54	±0.011	0	0
citronellal	0.20	±0.024	0	0	0	0	0.70	±0.050	0.29	±0.070	0	0
geranyl acetate	0.91	±0.012	0.59	±0.020	0	0	0	0	0	0	0	0
cis-geraniol	0	0	4.96	±0.033	0	0	0	0	0	0	0	0
trans-geraniol	0	0	3.39	±0.036	1.58	±0.017	0	0	0	0	0	0
Linalool	0.29	±0.063	0	0	0.63	±0.058	1.73	±0.059	1.51	±0.057	0	0
linalyl propanoate	0	0	0.25	±0.037	0	0	0.24	±0.039	0	0	0	0
bergamol	0	0	0	0	0	0	0	0	0	0	9.53	±0.071
neryl acetate	0.49	±0.061	0.42	±0.052	0	0	0.16	±0.047	1.14	±0.092	0.21	±0.081
*Oxygenated monocyclic monoterpenes*												
trans-carveol	0	0	0	0	3.36	±0.052	0	0	0	0	0	0
carvone	0	0	0	0	7.28	±0.032	0.10	±0.003	0	0	0	0
trans-p-2,8-mentadien-1-ol	0	0	0	0	1.84	±0.046	0	0	0	0	0	0
menthol	0,12	±0,006	0	0	0	0	0	0	0	0	0	0
menthone	0,06	±0,029	0	0	0	0	0	0	0	0	0	0
1-terpinen-4-ol	0	0	0	0	5.57	±0.075	0	0	0.42	±0.013	0	0
*Oxygenated bi- and tricyclic monoterpenes*												
pinene oxide	0.17	±0.016	0	0	0	0	0.65	±0.050	0	0	0	0
pinocarveol	0	0	0	0	5.44	±0.035	0	0	0	0	0	0
verbenol	0.08	±0.013	0	0	4.90	±0.024	0	0	0	0	0	0
trans-verbenol	0	0	0	0	1.50	±0.038	0	0	0	0	0	0
*Oxygenated monoterpenes*	*13.76*		*44.76*		*32.80*		*4.93*		*7.67*		*10.09*	

*SD* standard deviation

**Table 3:** Sesquiterpenes, oxygenated sesquiterpenes and others compounds in lemon essential oils

	ETJA		Vera-Nord		Avicenna-Oil		Dufti by Gies		Croce Azzurra		Aromatic Art	
	Area (%)	SD	Area (%)	SD	Area (%)	SD	Area (%)	SD	Area (%)	SD	Area (%)	SD
*Sesquiterpenes*												
α-bergamotene	0	0	0.95	±0.078	2.31	±0.130	0	0	0.89	±0.057	0	0
ß-bergamotene	0	0	0	0	0	0	0	0	1.54	±0.062	0	0
ß-bisabolene	0	0	0.48	±0.097	0	0	0	0	1.82	±0.058	0	0
α-cadinene	0	0	0	0	0	0	0.13	±0.011	0	0	0	0
ß-cadinene	0	0	0	0	0	0	0.09	±0.008	0	0	0	0
calarene	0	0	0	0	0	0	0.15	±0.011	0	0	0	0
caryophyllene	0	0	0.24	±0.078	0	0	0.29	±0.091	1.11	±0.041	0.12	±0.011
α-caryophyllene	0	0	6.09	±0.084	0	0	0	0	0.06	±0.031	0	0
ß-farnesene	0	0	0	0	0	0	0	0	0.06	±0.006	0	0
germacene B	0	0	0	0	0	0	0	0	0.38	±0.009	0	0
valencene	0	0	0	0	0	0	0.32	±0.008	0	0	0	0
*Sesquiterpenes*	*0*		*7.76*		*2.31*		*0.98*		*5.84*		*0.12*	
*Oxygenated sesquiterpenes*												
caryophyllene oxide	0	0	0	0	3.20	±0.051	0	0	0	0	0	0
farnesol	0	0	4.48	±0.058	0	0	0	0	0	0	6.12	±0.119
*Oxygenated sesquiterpenes*	*0*		*4.48*		*3.20*		*0*		*0*		*6.12*	
*Others*												
hexanoic acid	0	0	0	0	0	0	0	0	0	0	0.47	±0.132
hydrocinnamic acid	0	0	0	0	0	0	0	0	0	0	6.54	±0.698
ß-ionene	0	0	0	0	0	0	0	0	0	0	7.81	±0.414
isopropyl myristate	0	0	0	0	0	0	0	0	0	0	42.78	±0.422
1,1-dimethoxy-2-phenylopropane	0	0	0	0	0	0	0	0	0	0	0.36	±0.031
*Others*	*0*		*0*		*0*		*0*		*0*		*57.60*	

*SD* standard deviation

ETJA and Avicenna-Oil lemon essential oils contained 20 and 18 terpenes and terpenoides, respectively, and the greatest diversity of this group of compounds was observed in Croce Azzurra and Vera-Nord essential oils (23 compounds). Limonene was present in the tested essential oils in the largest amounts—in essential oils of the German company Dufti (63.2 %), ETJA (48.3 %), Avicenna-Oil (42.0 %) and the Italian company Croce Azzurra (38.5 %). The type and percentage composition of other compounds were also different in all tested lemon essential oils and were characteristic for particular producers. The following compounds were additionally present in the essential oil of the German company Dufti: β-pinene (14.3 %), β-myrcene (6.9 %), γ-terpinene (4.8 %) and α-pinene (3.6 %) (Fig. 3a, Table 1). In the ETJA essential oil, other main compounds were β-pinene (15.1 %), α-pinene (11.1 %), citral and its isomers (11.4 % in total), γ-terpinene (4.8 %) and β-myrcene (4.1 %) (Fig. 2a, Table 1). Avicenna-Oil included β-pinene (15.2 %), carvone (7.3 %), 1-terpinen-4-ol (5.6 %), pinocarveol (5.4 %) and verbenol (4.9 %) (Fig. 1b, Tables 1, 2). The lemon essential oil of the Italian company Croce Azzurra contained large amounts of β-pinene (20.0 %), β-felandrene (15.0 %), α-pinene (5.6 %) and β-myrcene (5.4 %) (Fig. 3b, Table 1). Citral and its isomers (35.1 % in total), limonene (23.4 %), oraz β pinene (8.9 %), cis-, trans-geraniol (8.4 % in total), caryophyllene (6.4 %), γ-terpinene (5.9 %) and farnesol (4.5 %) (Fig. 1a, Tables 1, 2, 3) were present in the Vera-Nord essential oil in the largest amounts. The main ingredient of the Aromatic Art essential oil was isopropyl myristate (42.8 %), β-ionene (7.8 %), hydrocinnamic acid (6.5 %) and 10 terpenes, and oxygenated terpenes—mainly limonene (22.4 %), bergamol (9.5 %), farnesol (6.1 %) and β-pinene (2.2 %) (Fig. 2b, Tables 1, 2, 3)—accounted for only 41.6 % of the oil composition. The large amount of fatty acid ester (isopropyl myristate) was the reason that not good separation and not symmetrical peaks were achieved in this case, because the chromatographic method typically used for the separation of terpenes and terpenoids (non-polar column) is useless for fatty compounds (Fig. 2b).

Despite the fact that all tested lemon essential oils contained limonene, their impact on the growth of the tested *Candida* yeasts was different. The Dufti essential oil, although contained the largest amounts of limonene, did not inhibit the growth of any of the tested yeast strains. The Croce Azzurra essential oil also proved to be ineffective as far as the inhibition of the growth of the tested strains is concerned. Therefore, the inhibitory effect of essential oils does not depend on the concentration of limonene but on the presence of other biologically active substances and the sensitivity of yeasts. *C. glabrata* and *C. tropicalis* strains are much less sensitive than the *C. albicans* strain. Thus, only some essential oils at higher concentrations exhibit the antifungal potential against these yeast strains. *C. glabrata* 33 and *C. glabrata* 35 strains were the most resistant to the tested lemon essential oils (none of the tested products inhibited their growth). The effects of the essential oil activity against the tested yeast strains are shown in Table 4.

**Table 4:** Inhibitory activity of lemon essential oil

Oil and concentration	Zones of inhibition (mm) ± SD		
	*C. albicans* 31	*C. tropicalis* 32	*C. glabrata* 38
ETJA 0.6 %	0	0	0
ETJA 1.0 %	0	0	0
ETJA 1.6 %	0	0	0
ETJA 2.0 %	0	0	0
ETJA 2.6 %	0	0	45.0 ± 0.0
ETJA 3.0 %	0	0	44.8 ± 0.2
Vera–Nord 0.6 %	44.8 ± 0.2	0	0
Vera–Nord 1.0 %	44.8 ± 0.2	0	0
Vera–Nord 1.6 %	44.8 ± 0.2	0	0
Vera–Nord 2.0 %	44.8 ± 0.2	0	0
Vera–Nord 2.6 %	45.0 ± 0.0	15.3 ± 0.4	0
Vera–Nord 3.0 %	45.0 ± 0.0	16.3 ± 0.4	0
Avicenna–Oil 0.6 %	23.0 ± 0.0	0	0
Avicenna–Oil 1.0 %	25.0 ± 0.7	0	0
Avicenna–Oil 1.6 %	44.8 ± 0.2	0	0
Avicenna–Oil 2.0 %	44.8 ± 0.0	0	0
Avicenna–Oil 2.6 %	44.8 ± 0.0	0	44.6 ± 0.4
Avicenna–Oil 3.0 %	45.0 ± 0.0	0	44.8 ± 0.2
Aromatic Art 0.6 %	0	0	0
Aromatic Art 1.0 %	0	0	0
Aromatic Art 1.6 %	0	0	0
Aromatic Art 2.0 %	0	0	0
Aromatic Art 2.6 %	0	0	0
Aromatic Art 3.0 %	17.4 ± 0.1	0	0
Dufti by Gies 0.6 %	0	0	0
Dufti by Gies 1.0 %	0	0	0
Dufti by Gies 1.6 %	0	0	0
Dufti by Gies 2.0 %	0	0	0
Dufti by Gies 2.6 %	0	0	0
Dufti by Gies 3.0 %	0	0	0
Croce Azzurra 0.6 %	0	0	0
Croce Azzurra 1.0 %	0	0	0
Croce Azzurra 1.6 %	0	0	0
Croce Azzurra 2.0 %	0	0	0
Croce Azzurra 2.6 %	0	0	0
Croce Azzurra 3.0 %	0	0	0

*SD* standard deviation

The tests show that essential oils containing larger amounts of oxygenated monoterpenes have much better antimicrobial properties. Vera-Nord and Avicenna-Oil essential oils contain 44.8 and 32.8 % of monoterpenoids, respectively, and most effectively inhibit the growth of the *Candida* strain across the full range of the concentrations used. The measured zones of inhibition for *Candida albicans* 31, depending on the concentration of these essential oils, were from 23 mm to complete inhibition—45 mm (Table 4). Furthermore, it may be concluded that antifungal properties are observed only in the case of essential oils containing more than 10 % of oxygenated monoterpenes. With the lower content of monoterpenoids, the inhibition of the *Candida* strain growth was observed only for the highest concentrations when ETJA and Aromatic Art essential oils were used. The Aromatic Art essential oil inhibited the growth of *C. albicans* only at the highest concentration of 3 %, and the zone of inhibition measured approximately 17 mm. The growth of *C. glabrata* 38 was inhibited by ETJA and Avicenna-Oil essential oils (approximately 45 mm) at the concentration of 2.6 and 3 %. The *C. tropicalis* 32 strain was sensitive only to the Vera-Nord essential oil at concentrations of 2.6 and 3 %. The zones of inhibition measured 15.3 mm (± 0.40) and 16.3 mm (± 0.40), respectively.

Not only the high content of monoterpenoids but also the presence of particular active ingredients and maintaining adequate proportions between these ingredients is necessary for lemon essential oils to exhibit antimicrobial properties. High antimicrobial activity of the Vera-Nord essential oil is probably due to cis- and trans-citral isomers present in this essential oil in the largest amounts as compared to all essential oils tested. Additionally, the activity of citral present in this essential oil is enhanced by isomers of geraniol and γ-terpinene. All these compounds are known for their fungicidal properties. Antimicrobial properties of Avicenna-Oil and ETJA essential oils may be due to the synergistic effect of citral and verbenol. However, the proportions of these terpenoides are different depending on the oil, namely, the ETJA essential oil contained 11.4 % of citral isomers and trace amounts of verbenol (0.1 %). The proportions of these ingredients in the Avicenna-Oil essential oil are reversed: it contains small amounts of citral (0.7 %) and quite significant amounts of verbenol (6.4 %). Antifungal potential may be enhanced by terpenoides, such as carvone (7.3 %) or 1-terpinen-4-ol (5.6 %), present in the Avicenna-Oil essential oil.

The chemical composition of the Aromatic Art lemon essential oil differs considerably from the chemical composition of other essential oils tested. This oil inhibits the growth of *C.* *albicans* C31 only in small degree.

The MIC of lemon essential oil causing total growth inhibition was determined only for *C. albicans* 31 in the case of Vera-Nord and Avicenna-Oil essential oils. The obtained MIC value was 0.6 %, the minimum concentrations of essential oils used. Other essential oils reduced the survival rate of the selected *Candida* strains by 50–98 %.

## Discussion

Reports on the antifungal potential of lemon essential oil against *Candida* yeasts are ambiguous [5, 7, 8]. Devkatte et al. [7] obtained the MIC value of 0.5 % and the average zone of inhibition of approximately 17.5 mm for the lemon essential oil tested by them, while the zones of inhibition for the selected *Candida* strains determined by Warnke et al. [8] measured 16–43 mm. Higher mean values related to the zones of inhibition for *C. albicans,* ranging from 23 to 45 mm, may be due to the differences in diffusion of particular essential oil ingredients in agar [7, 9] and the concentration of biologically active substances, mainly citral, geraniol and verbenol. Thus, when evaluating the essential oils produced from a particular plant, it is good to know their chemical composition. According to many authors, the essential oil volatile phase composition depends on the conditions in which the plant has grown, its development stage and the way it is stored [9]. In the case of citrus fruits, climatic conditions and the region of origin of the fruit are important [11].

Limonene—a monocyclic terpene—was the main ingredient of the tested lemon essential oils. However, the antimicrobial activity depended on the content of oxygenated monoterpenes—the higher the content, the better fungicidal effects were observed. This is confirmed by the literature data [11, 13]. Vera-Nord and Avicenna-Oil essential oils contain 44.8 and 32.8 % of oxygenated monoterpenes, respectively, and exhibit antifungal potential against *C. albicans* across the full range of the concentrations used and may be used as antifungal preparations against this yeast strain. The Aromatic Art essential oil, which is less effective as far as the inhibition of the growth of *C. albicans* is concerned, contains hydrocinnamic acid and bergamol—compounds known for their antimicrobial properties [9, 11].

*Candida glabrata* and *Candida tropicalis* strains were less sensitive to the tested essential oils than the *C. albicans* strain—their growth was inhibited by essential oils used at higher concentrations. The Vera-Nord essential oil contains significant amounts of cis- and trans-citral isomers or geraniol and γ-terpinene known for their fungicidal properties [9, 11].

The growth of *C. glabarata* 38 was inhibited by two lemon essential oils: Avicenna-Oil and ETJA, which both contains citral and verbenol. Essential oils exhibit antifungal properties when they contain, at the same time, large amounts of one of these oxygenated monoterpenes and small amounts of the second one.

The tested essential oils contained also other valuable ingredients, such as carvone, 1-terpinen-4-ol and γ-terpinene—all of these compounds also exhibit antifungal potential, which has already been described in the literature [8, 9, 11, 13].

The use of lemon essential oils in all kinds of candidiasis seems to be an interesting solution because of their documented safety. It should also be noted that some of the authors at the same time emphasise that it is necessary to conduct tests on the toxicity and possible allergenic effects of selected essential oils [7]. Therefore, understanding the relationship between the chemical composition of essential oils and their antimicrobial activity is of great importance due to the potential use of lemon essential oil-based products as natural remedies against candidiasis caused by *C. albicans*.

## Conclusions

- Lemon essential oils with high content of monoterpenoids may be an ingredient of products against candidiasis caused by *Candida.*
- Avicenna-Oil and ETJA contain citral and verbenol. Large amounts of one of these oxygenated monoterpenes and small amounts of the second one, at the same time, may be the reason for the growth inhibition of the tested *C. glabarata* yeasts.
- Antifungal potential of the Aromatic Art essential oil at higher concentrations is probably the result of the synergistic interaction between hydrocinnamic acid and bergamol.
